# Validation of the 8^th^ edition UICC/AJCC TNM staging system for HPV associated oropharyngeal cancer patients managed with contemporary chemo-radiotherapy

**DOI:** 10.1186/s12885-019-5894-8

**Published:** 2019-07-09

**Authors:** Kirsten van Gysen, Mark Stevens, Linxin Guo, Dasantha Jayamanne, David Veivers, Andrew Wignall, Leo Pang, Alexander Guminski, Adrian Lee, George Hruby, Paula Macleod, Alon Taylor, Thomas Eade

**Affiliations:** 1 Department of Radiation Oncology, Northern Sydney Cancer Centre, Royal North Shore Hospital, St Leonards, NSW 2065 Australia; 2 Department of Medical Oncology, Northern Sydney Cancer Centre, Royal North Shore Hospital, St Leonards, NSW Australia; 3 Head and Neck Surgery, Royal North Shore Hospital, St Leonards, NSW Australia; 4 Bill Walsh Translational Cancer Research Laboratory, University of Sydney, Sydney, NSW Australia; 5 Northern Clinical School, Sydney Medical Program, University of Sydney, Sydney, NSW Australia

**Keywords:** Human papilloma virus, HPV, Oropharyngeal carcinoma, Staging, TNM, Radiotherapy

## Abstract

**Background:**

To compare outcomes of high-risk human papilloma virus-related oropharyngeal squamous cell carcinoma (HPV OPSCC) treated with modern radiation treatment (RT) and daily image-guidance, staged with the 7^th^ versus the 8^th^ Edition (Ed) Union for International Cancer Control (UICC)/American Joint Committee on Cancer (AJCC) TNM staging systems.

**Methods:**

All eligible patients with HPV OPSCC treated definitively over a 10-year period (2007–2016) at a single institution were included. Protocols consisting of either RT or chemo-radiation (CRT) (weekly cisplatin or cetuximab) +/− neoadjuvant chemotherapy for those with bulky disease were used. All patients were Fluorine-18-deoxyglucose positron emission tomography (FDG-PET) staged at baseline and at intervals for up to 2 years post-treatment. Patients received parotid-sparing intensity modulated or volumetric modulated arc therapy with simultaneous integrated boost to either 70Gy in 35 fractions or 66Gy in 30 fractions. The overall survival (OS) was determined for each stage using the 7^th^ Ed and subsequently with the updated 8^th^ Ed staging system.

**Results:**

One hundred fifty-three patients were analysed. Patient stage groupings varied between the 7^th^ and 8^th^ Eds respectively; Stage I (0.7% vs 64.7%), Stage II (8.5% vs 22.2%), stage III (21.6% vs 12.4%) and stage IV (69.3% vs 0.7%). In the 7^th^ Ed, the 5 year probability of OS for stages I to III was 90%, versus stage IV 85.5%. There was no statistically significant difference between the staging groups (*p* = 0.85). In the 8^th^ Ed there was a statistically significant difference in 5 year OS for stage I and stage II disease (96.9% vs 77.1% respectively; *p* < 0.0001), but not between stage II and III disease (*p* = 0.98).

**Conclusions:**

The new 8^th^ Ed UICC/AJCC TNM staging system better discriminates between stage I and Stage II HPV OPSCC with respect to OS compared with the 7^th^ Ed staging system. Further investigation is required for stage III or IV patients.

## Background

The last decade has seen a rise in the cumulative incidence of oropharyngeal squamous cell carcinoma (OPSCC), particularly in white, middle-aged males with moderate alcohol and limited smoking exposure. Epidemiologically this is due to the increased incidence of high-risk HPV-related cancers (HPV OPSCC) [1, 2]. HPV-positive tumour phenotype is the single most favourable non-anatomic prognostic factor for OPSCC outcome. HPV OPSCC are highly responsive to treatment with significantly improved disease-specific and overall survival, with lower rates of loco-regional failure and death without failure [3]. HPV OPSCC however has a predilection for early regional lymph node metastases which adversely skews anatomic-based staging classification. Approximately 80% of HPV OPSCC patients are thus classified with advanced (stage IV) disease when using the AJCC 7^th^ Ed staging manual [4, 5]. Paradoxically HPV OPSCC have significantly better outcomes than HPV-negative stage IV OPSCC. The latter being more commonly associated with significant smoking (> 10 pack-years) and alcohol exposure [3].

According to Groome at al [6] a staging system should stratify so that there is similar survival for each patient subgroup (hazard consistency), and should discriminate between different subgroups (hazard discrimination). The assigned stage should also estimate prognosis (i.e. have a high predictive ability) and reliably select an appropriate treatment plan (or clinical trial) for an individual patient. Finally stage groupings should be proportionally balanced to facilitate statistical analysis, clinical trial planning, and audit. The 7^th^ Ed UICC/AJCC TNM classification of OPSCC adequately reflects the behaviour of “traditional” head and neck cancers associated with tobacco and alcohol abuse. However its development (2003–2009) preceded much of the emerging knowledge of the HPV OPSCC phenotype and as a staging tool the 7^th^ Ed UICC/AJCC Manual is poorly predictive of the clinical outcomes for this group.

The recently released 8^th^ Ed of the UICC/AJCC TNM Staging Manual has recognised the prognostic power of high-risk HPV cancer status and has attempted to “bridge the gap” from a population-based anatomic model of disease to a more personalised approach [7]. The 8^th^ Ed has adopted the changes in T and N categories proposed by the International Collaboration on Oropharyngeal cancer Network for Staging (ICON-S). This multicentre cohort study retrospectively examined the outcomes of 1907 non-metastatic (M0) HPV OPSCC patients treated in Europe and North America [8, 9]. The ICON-S staging system has been externally validated by an Australian group in 279 historical patients [10] and two further studies have retrospectively validated the 8^th^ Edition TNM staging classification in treated German [11] and Japanese [12] HPV OPSCC populations.

To date however the HPV OPSCC patients selected to validate the 8^th^ Ed Staging Manual have generally received either three-dimensional conformal radiotherapy (3DCRT) or early iterations of intensity-modulated radiation therapy (IMRT). Systemically-dosed three weekly cisplatin rather than low-dose weekly cisplatin was also commonly used in these protocols. A Surveillance, Epidemiology, and End Results (SEER) database study of 3172 patients reported significant improvement in cancer specific survival (CSS) and reduced toxicity in head and neck cancer patients treated with IMRT compared with those treated with non-IMRT techniques [13]. We have previously published our IMRT/VMAT (volumetric modulated arc therapy) outcomes for our unit and confirmed excellent survival in our HPV OPSCC population using low-dose weekly cisplatin or neoadjuvant TPF (docetaxel, cisplatin, fluorouracil) followed by weekly low-dose cisplatin or cetuximab in those patients with initial bulky (T4 or N3) disease [14].

In this study we aim to further validate the major T and N changes proposed by the 8^th^ Ed UICC/AJCC TNM Cancer Staging Manual in a group of patients treated with contemporary RT using parotid-sparing IMRT/VMAT and image guidance with concurrent cisplatin given weekly.

## Methods

### Study population

This study was a retrospective analysis from a prospectively collected, ethics approved, head and neck database of all newly diagnosed patients from 2006 to 2016 at Royal North Shore Hospital, Sydney, Australia. All patients with biopsy-confirmed, loco-regionally confined p16+ OPC treated with curative intent, with either RT or CRT, were included. Patients with T4 disease or initial bulky lymphadenopathy received neoadjuvant TPF chemotherapy and were also included. All patients were assessed and staged at baseline at a Head and Neck multidisciplinary meeting (MDM), attended by head and neck surgeons, radiation oncologists, medical oncologists, pathologists, radiologists and nuclear medicine physicians. All patients were examined clinically and with nasoendoscopic examination and their pathology was reviewed. After diagnosis, all patients were staged with a baseline whole body FDG-PET/contrast enhanced computed tomography (CT) which was subsequently reviewed at the MDM. Magnetic resonance imaging (MRI) was performed on patients where local extension of tumour needed evaluation. Staging, as per the 7^th^ Ed AJCC TNM was assigned at the MDM, with all information being entered onto a real-time database.

### P16 immunohistochemistry

HPV-mediated carcinogenesis was determined by immunohistochemical (IHC) testing (CINtec p16 antibody) for over-expression of p16. A positive test was defined as diffuse (> 75%) tumour expression with at least moderate intensity staining, localised to both the cytoplasm and the nucleus [15].

### 8^th^ edition UICC/AJCC TNM staging system

In the 8^th^ Ed, T category remains the same, except for the removal of Cis and the category T4b; however, the nodal and group categories have been reclassified. Patients with clinically involved lymph nodes, ipsilateral and less than 6 cm in size, whether individual or multiple nodes involved, are now categorised as N1. Contralateral or bilateral lymph nodes, less than 6 cm in size, are classified as N2. Lymph nodes greater than 6 cm indicate worst survival from regional disease and are category N3. Staging groups are as follows: stage I (T1–2,N0–1), stage II (T1–2N2, T3 N0–2), stage III (T4 or N3), stage IV (M1) [8].

### Treatment

Patients were treated as per standard department protocol with parotid sparing IMRT or VMAT as previously described [14]. Patients with T1 N0 and T2 N0 were treated with RT alone using a dose/fractionation schedule of 66Gy in 30 daily fractions [16]. All patients with node positive disease received concurrent CRT with 70Gy in 35 daily fractions and either weekly cisplatin (40 mg/m^2^) or cetuximab (loading dose 400 mg/m^2^, then 250 mg/m^2^ weekly from cycle 2). Those with bulky nodal disease received three cycles of neoadjuvant TPF chemotherapy followed by concurrent CRT with 70Gy in 35 fractions and either weekly cisplatin or cetuximab. Patients with well-lateralised tumours of the tonsil (> 1 cm from midline, T1–2) and low nodal burden (N2a, solitary ipsilateral node, < 3 cm) were recommended ipsilateral neck RT. All other oropharyngeal tumours were treated with bilateral neck irradiation.

All volumes and plans were reviewed at a weekly head and neck planning meeting to ensure adequate tumour coverage, protocol adherence and plan quality. Patients were reviewed weekly during treatment to manage acute toxicity and in the weeks following treatment if significant toxicity continued. A restaging PET scan was performed 12 weeks post completion of treatment to assess for metabolic response [17]. Patients with residual PET-avid disease or an inconclusive scan underwent a repeat scan at 16 weeks to assess for resolution of changes. If the 16 week scan remained positive, these patients were considered for biopsy and potential salvage surgery.

### Statistical considerations

**Fig. 1:**
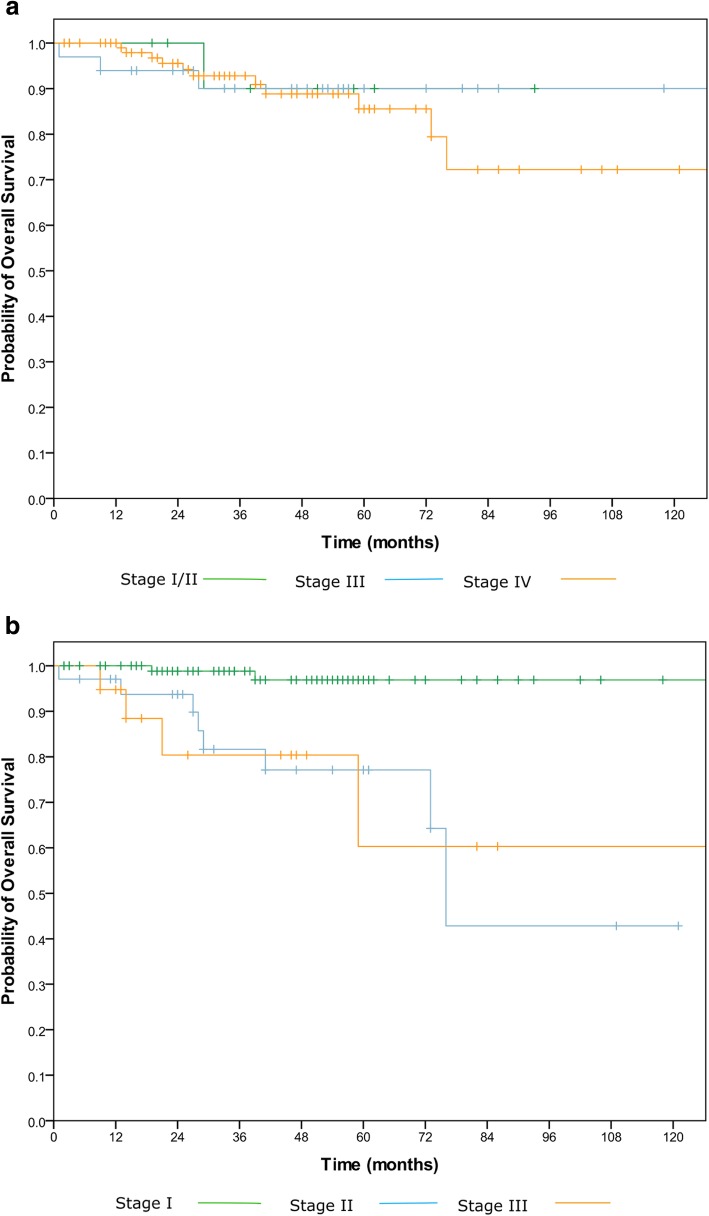
a and b (left to right): Kaplan Meier curves demonstrating probability of overall survival of patients with HPV associated oropharyngeal cancer according to **a** 7^th^ Edition TNM staging system with no significant difference between any stage groups and **b** 8^th^ Edition TNM staging system with a statistically significant difference between stage I and II (*p* < 0.0001), but not between stages II and III. The 5 year probability of overall survival was 96.9% for stage I, 77.1% for stage II and 60.3% for stage III

**Fig. 2:**
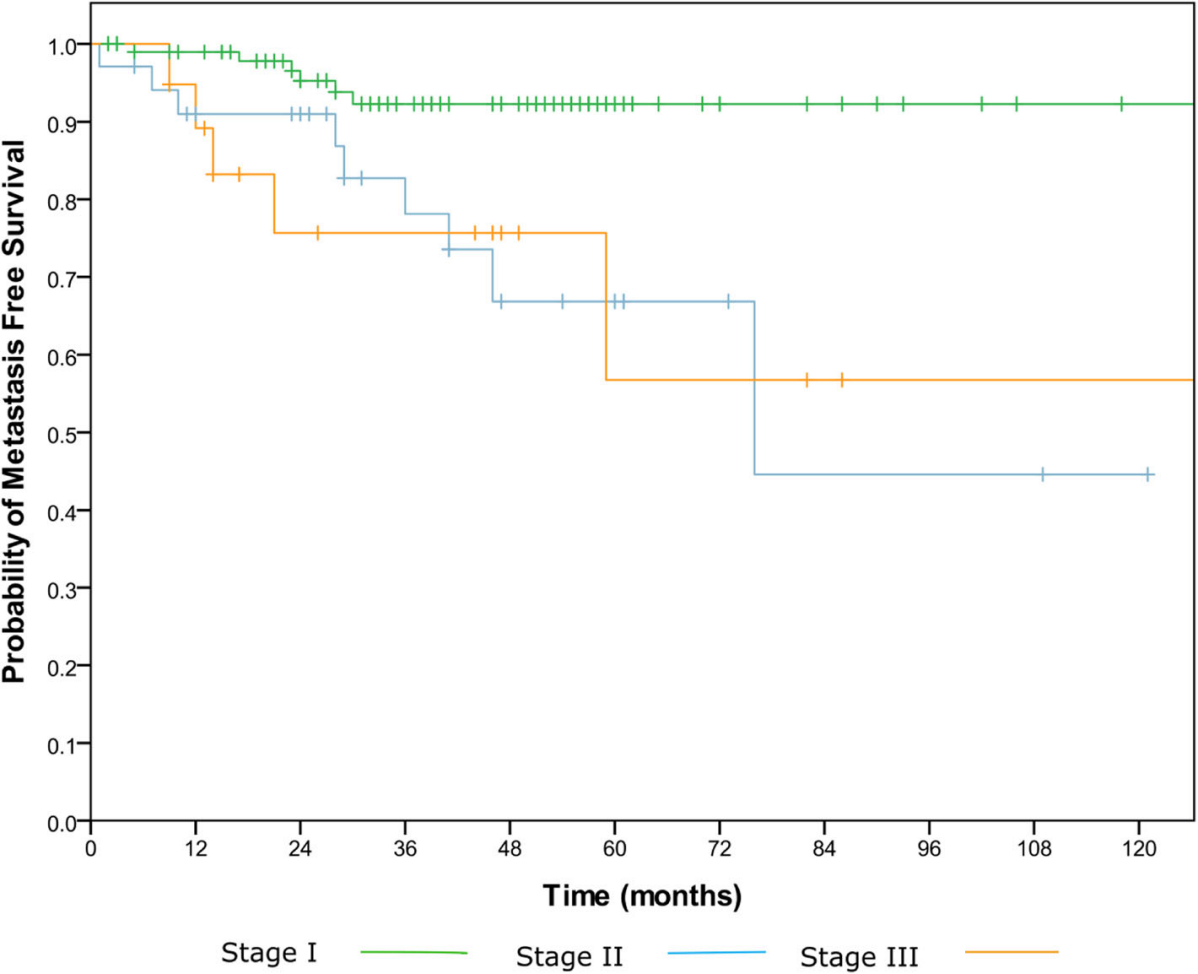
Kaplan Meier curve demonstrating probability of metastasis free survival in patients with HPV associated oropharyngeal cancer according to 8^th^ Ed TNM staging system with a statistically significant difference between stages I and II (*p* < 0.002), but not between stages II and III

OS curves (Fig. 1a and b) were calculated using the Kaplan-Meier method for the 7^th^ and 8^th^ Ed TNM staging system with 95% confidence intervals (CI). OS was calculated from the start of treatment to the date of death from any cause or the last date on which the patient was known to be alive. Within each staging classification, the group stages were compared using the log rank test. The 5 year OS were calculated and reported by stage. Metastasis-free survival curves (Fig. 2) were calculated using the Kaplan-Meier method for the 8^th^ edition TNM staging system.

To identify survival differences between stages for either staging classification, and account for potential factors of age, addition of systemic therapy, and smoking history, Cox regression analysis was performed and hazard ratios (HR) with 95% CI were reported for each variable. Age was assessed as a categorical variable (≤60 years vs > 60 years). Smoking was assessed in the categories of non-smoker, ≤10 pack/years and > 10 pack years. Systemic therapy was assessed individually, including neoadjuvant chemotherapy, concurrent cisplatin and cetuximab.

IBM SPSS Statistics version 24 was used in the data analysis.

## Results

### Patient and tumour characteristics

A total of 153 patients with HPV OPSCC were included. The median follow up was 40.5 months. The median age was 59 years (range, 31–82) and 88.9% of the cohort was males, 44% were non-smokers. Of the smokers, 16.3% had less than 10 pack year smoking history, and 17.6% had accumulated more than 20 pack years. The primary was in the tonsil in 60.2% of cases and in the base of tongue in 35.9%.

### Treatment

74.6% of patients received systemic therapy, with 68.1% with weekly cisplatin. Neoadjuvant TPF was used in 14 patients (7^th^ Ed: All 14 in stage IV disease, 8^th^ Ed: 3 in stage I disease (3%), 4 in stage II disease (11.7%), 7 in stage III disease (36.8%)).

### Stage

Patient stage groupings varied between the 7^th^ and 8^th^ Eds across all stage groups (see Table 1); Stage I (0.7% vs 64.7%), Stage II (8.5% vs 22.2%), stage III (21.6% vs 12.4%) and stage IV (69.3% vs 0.7%). T category was T1 or T2 in 74.2% of patients. In the 7^th^ edition, 39.2% of patients were N2b (multiple ipsilateral nodes < 6 cm). In the 8^th^ edition, 66.9% of patients were classified as N1 (ipsilateral nodes, < 6 cm) (see Table 2). The predominant stage shift was from 7^th^ Ed stage IV to 8^th^ Ed stage I by 41.2% of patients as seen in Table 3. The next most frequent shifts were from 7^th^ Ed stage III to 8^th^ Ed Stage I and from 7^th^ Ed stage IV to 8^th^ Ed stage II, with 15.7% of patients in each case.

**Table 1:** Number (and %) of patients in each stage group as defined by the 7th and 8th Edition TNM staging systems

Stage	7^th^ Ed TNM. Number (%)	8^th^ Ed TNM Number (%)
I	1 (0.7%)	99 (64.7%)
II	13 (8.5%)	34 (22.2%)
III	33 (21.6%)	19 (12.4%)
IV	106 (69.3%)	1 (0.7%)
Total	153	153

**Table 2:** Patient and tumour characteristics

Characteristic	Number (%)
Age	Median: 59
	Range: [31, 82]
Sex	Male: 136 (88.9%)
	Female: 17 (11.1%)
Smoking status	Non-smoker: 68 (44.4%)
	< =10 Packs/year: 25 (16.3%)
	> 10–20 Packs/year: 18 (11.8%)
	> 20 Packs/year: 27 (17.6%)
	Unknown: 15 (9.8%)
Primary site	Tonsil: 92 (60.2%)
	Base of tongue: 55 (35.9%)
	Oropharynx: 1 (0.7%)
	Other: 5 (3.2%)
T stage 7^th^ Ed/8^th^ Ed	T1: 47 (30.7%)
	T2: 67 (43.8%)
	T3: 29 (19.0%)
	T4a: 9 (5.9%)
	T4b: 1 (0.7%)
N stage 7^th^ Ed	N0: 17 (11.1%)
	N1: 27 (17.6%)
	N2a: 16 (10.5%)
	N2b: 60 (39.2%)
	N2c: 28 (18.3%)
	N3: 5 (3.3%)
N stage 8^th^ Ed	N0: 17 (11.1%)
	N1: 103 (66.9%)
	N2: 28 (18.3%)
	N3: 5 (3.3%)
Systemic therapy	Nil: 39 (25.4%)
	Weekly Cisplatin: 85 (55.6%)
	Cetuximab: 10 (6.5%)
	Neoadjuvant TPF: 14 (9.2%)

**Table 3:** The number (and %) of patients migrating by stage from the 7^th^ to the 8^th^ edition TNM staging system

	Stage	8^Th^ Edition TNM			
		I	II	III	IV
7^th^ Edition TNM	I	1 (0.7%)			
	II	11 (7.2%)	2 (1.3%)		
	III	24 (15.7%)	8 (5.2%)	1 (0.7%)	
	IV	63 (41.2%)	24 (15.7%)	18 (11.8%)	1 (0.7%)

### Survival

#### 7^th^ Ed TNM staging

In the 7th Ed, the 5 year probability of OS (Fig. 1a) for stage I/II or III was 90%, and stage IV 85.5%. There was no OS difference between the staging groups (*p* = 0.85).

#### 8^th^ Ed TNM staging

There was a statistically significant difference between 5 year OS (Fig. 1b) for stage I and stage II disease (96.9% vs 77.1% respectively) (*p* < 0.0001). Stage III patients had 60.3% probability of OS at 5 years, there was no significant difference between stage II and stage III disease (*p* = 0.98).

### Metastatic disease

The 5 year probability of metastasis free survival according to the 8^th^ Ed TNM staging was 92.3% for stage I, 66.8% for stage II and 56.7% for stage III. There was a statistically significant difference between stage I and II ((*p* < 0.002), but again no difference between stage II and III (Fig. 2).

### Multivariate analysis

Age did not have an impact on survival (HR = 0.431, 95% CI = [0.153, 1.214], *p* = 0.111). Smoking pack years, when divided into ≤10 pack years and > 10 pack years had no effect on OS (HR = 2.18, 95% CI = [0.363, 13.074], *p* = 0.394). There was no difference found between use of either neoadjuvant TPF, or concurrent cisplatin or cetuximab and their impact on OS. (TPF vs Cisplatin: HR = 1.411, 95% CI = [0.308, 6.462], *p* = 0.658; Cetuximab vs Cisplatin: HR = 1.807, 95% CI = [0.221, 14.815], *p* = 0.581).

## Discussion

The 8^th^ Ed UICC/AJCC TNM staging system recognises HPV OPSCC as a separate disease entity. While there have been challenges associated with adapting an anatomical classification system to incorporate personalised biological markers, the aim has been to more accurately reflect the superior survival outcomes that are seen in these patients, improve prognostication and potentially guide treatment decisions. We used our single institution data to validate the utility of the new staging system in a cohort of patients treated with image guided IMRT/VMAT and (predominantly) weekly cisplatin chemotherapy.

The updated 8^th^ Ed UICC/AJCC TNM staging system classifies the majority of HPV OPCC patients into stage I or stage II disease. In contrast 69.3% of our cohort was stage IV in the 7^th^ Ed, with 86.9% of patients being down-staged to stage I or II in the 8^th^ Ed. Similar results have been reported in previous validation series, which have all demonstrated a large migration from stage IV to stage I or II disease [10–12]. One difference in our analysis were the low numbers of new stage III patients (12.4%) as compared to previously published series (23.3% in the Brisbane series and 20.7% in a German study) [10, 11]. In the 8^th^ Ed, stage III is defined as either N3 disease or cT4 disease. Of our 153 patients, only 3.3% were N3 and 6.6% were T4. Our cohort were also more likely to be light (< 10 pack year) or never smokers (60.7%) and from higher socio-economic groups. According to the 2011 Census from the Australian Bureau of Statistics, our Area Health Service was recorded as the most advantaged Local Government Area in the state of New South Wales, and the second most privileged in Australia overall [18]. Our better-educated, higher socio-economic status patients thus may have been more likely to present prior to developing bulky neck disease. Alternatively our definition of N3 disease may have been influenced by the staging FDG - PET. The latter identified individual nodal avidity even in patients with clinically confluent masses. It was our MDM group consensus to N stage individual lymph nodes within distinct neck levels on FDG-PET even when total lymph node mass may have measured > 6 cm.

Our results confirm that in patients treated with contemporary RT, and predominantly de-escalated chemotherapy, the 8^th^ Ed staging system demonstrated a statistically significant difference in OS between stage I and stage II patients. There was no difference however seen for stage II versus stage III patients. This may be reflective of a chance effect due to the low number (*n* = 19) of stage III patients or alternatively improved real outcomes due to treatment intensification (e.g. neoadjuvant TPF followed by CRT) rather than a failure of the new staging system.

A previous Australian group included 279 HPV OPSCC patients with a median follow up of 62 months and 3rd weekly chemotherapy [10]. At 3 years, their results demonstrated a significant OS difference between stages I and II, but no initial difference in either OS or loco-regional control between stages II and III; significant divergence became evident with longer follow up, supporting the hypothesis that failures in HPV OPSCC tend to be both distant (rather than local) and delayed [19]. A German validation study examined a cohort of 150 HPV OPSCC patients treated predominantly with upfront surgery followed by adjuvant risk-adapted (C)RT [11]. Those treated with definitive CRT, received either three weekly cisplatin or a combination of fluorouracil and mitomycin C. Despite this treatment heterogeneity, our comparative end-results demonstrated very similar conclusions; a significant OS difference between stages I and II, but not stages II and III. Our reported 5 year OS rates were comparable for each stage however (stage I: 96.9% vs 94.4%, stage II: 77.1% vs 77.5 and stage III: 60.3% vs 63.9%). A Japanese validation study [12] of 111 patients with HPV OPSCC, with a median follow up of 41 months and treated predominantly with CRT with either platinum or docetaxel based chemotherapy, demonstrated a statistical difference in OS between stages II and III, but not between stages I and II when using the 8^th^ Ed. They reported 3 year OS as 90.9% for stage I and II and 70.2% for stage III. The authors of these four separate analyses agreed that the 8^th^ Ed was a better discriminator of OS in HPV OPSCC than the 7^th^ Ed, however, the variation in results between these studies suggests that further refinement of the 8^th^ Ed is required, or that larger validation project is needed.

HPV-mediated carcinogenesis in the current study was confirmed by immunohistochemistry testing for diffuse nuclear-pattern over-expression of the tumour suppressor protein p16, a cyclin-dependent kinase 2A [15]. In those patients with high-risk HPV OPSCC, the rate of HPV negativity has been reported to be between 5 and 20%, due to amplification of p16 unrelated to HPV, usually secondary to epigenetic or genetic silencing [3, 20]. In a publication from the Netherlands, 12.4% of 388 HPV OPSCC were HPV-DNA negative and demonstrated significantly worse 5 year OS compared with HPV positive tumours [21]. This reflects previously reported data that patients with co-tested p16+/ HPV- tumours had worse overall survival compared with p16+/ HPV+ disease [22]. While these patients represent a small proportion of the patient population, it is worth considering their impact on the performance of the 8^th^ Ed TNM staging system in future studies.

There are a number of limitations to this study. The small numbers of stage III patients, and the limited number of patients treated with neoadjuvant chemotherapy make it difficult to make any conclusions regarding HPV OPSCC and the role of upfront chemotherapy.

Due to their excellent response to RT, a number of studies are investigating the potential to de-escalate HPV OPSCC treatment, with the goal of maintaining disease control, but reducing morbidity. The current standard of care in HPV OPSCC remains radiotherapy with concurrent cisplatin, as two recently published randomised trials comparing the use of cisplatin and cetuximab concurrently with radiotherapy in HPV OPSCC reported worse tumour control with cetuximab and no benefit in terms of toxicity [23, 24]. The potential to de-escalate however remains a pertinent question for clinical trials, but it should be noted that the excellent outcomes in many stage I patients were achieved with combined modality therapy. Our data supports relative de-escalation with weekly instead of 3rd weekly cisplatin dosing, particularly in new stage I and II disease patients.

## Conclusion

The 8^th^ Ed UICC/AJCC TNM staging system appears to better stratify early stage HPV OPSCC patients with regard to OS following definitive treatment with contemporary RT and daily image guidance compared with the 7^th^ Ed staging system. Further investigation is required to better understand the stage III cohort and to determine which patients may be suitable for de-escalated treatment.

## Data Availability

The datasets used and analysed during the current study are available from the corresponding author on reasonable request.

## Abbreviations

3DCRT: Three-dimensional conformal radiotherapy
AJCC: American Joint Committee on Cancer
CI: Confidence intervals
CRT: Chemo-radiation
CSS: Cancer specific survival
CT: Computed tomography
Ed: Edition
FDG-PET: Fluorine-18-deoxyglucose positron-emission tomography
HPV OPSCC: Human papilloma virus-related oropharyngeal squamous cell carcinoma
ICON-S: International Collaboration on Oropharyngeal cancer Network for Staging
IHC: Immunohistochemical
IMRT: Intensity-modulated radiation therapy
MDM: Multidisciplinary meeting
MRI: Magnetic resonance imaging
OPSCC: Oropharyngeal squamous cell carcinoma
OS: Overall survival
RT: Radiation treatment
SEER: Surveillance, Epidemiology, and End Results
TPF: Docetaxel, cisplatin, fluorouracil
UICC: Union for International Cancer Control
VMAT: Volumetric modulated arc therapy
